# Study protocol of the German Study on Tobacco Use (DEBRA): a national household survey of smoking behaviour and cessation

**DOI:** 10.1186/s12889-017-4328-2

**Published:** 2017-05-02

**Authors:** Sabrina Kastaun, Jamie Brown, Leonie S. Brose, Elena Ratschen, Tobias Raupach, Dennis Nowak, Constanze Cholmakow-Bodechtel, Lion Shahab, Robert West, Daniel Kotz

**Affiliations:** 1 Institute of General Practice, Addiction Research and Clinical Epidemiology Unit, Medical Faculty of the Heinrich-Heine-University Düsseldorf, Werdener Str. 4, 40227 Düsseldorf, Germany; 2 Research Department of Clinical, Educational and Health Psychology, University College London, London, United Kingdom; 3 Institute of Psychiatry, Psychology and Neuroscience, King’s College London, London, United Kingdom; 4 Department of Health Sciences, University of York, York, United Kingdom; 5 Department of Epidemiology and Public Health, University College London, London, United Kingdom; 6 Department of Cardiology and Pneumology, University Medical Center Göttingen, Göttingen, Germany; 7 Institute and Outpatient Clinic for Occupational, Social and Environmental Medicine, Clinical Centre of the Ludwig Maximilian University Munich, Munich, Germany; 8 Comprehensive Pneumology Center, Munich, Germany; 9 German Center for Lung Research, Giessen, Germany; 10 Public Health and Epidemiology, Clinical and Real World Research, Kantar Health GmbH, Munich, Germany; 11 Department of Family Medicine, CAPHRI School for Public Health and Primary Care, Maastricht University, Maastricht, The Netherlands

**Keywords:** German Study on Tobacco Use, Epidemiology, Tobacco smoking, E-Cigarette, Household Survey

## Abstract

**Background:**

The prevalence of tobacco smoking in Germany is high (~27%). Monitoring of national patterns of smoking behaviour and data on the “real-world” effectiveness of cessation methods are needed to inform policies and develop campaigns aimed at reducing tobacco-related harm. In England, the Smoking Toolkit Study (STS) has been tracking such indicators since 2006, resulting in the adaptation of tobacco control policies. However, findings cannot be directly transferred into the German health policy context. The German Study on Tobacco Use (DEBRA: “Deutsche Befragung zum Rauchverhalten”) aims to provide such nationally representative data.

**Methods/Design:**

In June 2016, the study started collecting data from computer-assisted, face-to-face household interviews in people aged 14 years and older. Over a period of 3 years, a total of ~36,000 respondents will complete the survey with a new sample of ~2000 respondents every 2 months (=18 waves). This sample will report data on demographics and the use of tobacco and electronic (e-)cigarettes. Per wave, about 500–600 people are expected to be current or recent ex-smokers (<12 months since quitting). This sample will answer detailed questions about smoking behaviour, quit attempts, exposure to health professionals’ advice on quitting, and use of cessation aids. Six-month follow-up data will be collected by telephone.

**Discussion:**

The DEBRA study will be an important source of data for tobacco control policies, health strategies, and future research. The methodology is closely aligned to the STS, which will allow comparisons with data from England, a country with one of the lowest smoking prevalence rates in Europe (18%).

**Trial registration:**

This study has been registered at the German Clinical Trials Register (DRKS00011322) on 25th November 2016.

**Electronic supplementary material:**

The online version of this article (doi:10.1186/s12889-017-4328-2) contains supplementary material, which is available to authorized users.

## Background

In Germany, 110,000 people die from smoking tobacco each year [1]. In total, 13% of the country’s mortality is attributable to smoking [1]. While the implementation of various tobacco control policies has led to a reduction in smoking prevalence in high-income countries such as in England (current prevalence = 18%) [2], the prevalence in the German adult population is still high (27%) [3]. Like in other countries, smoking prevalence is considerably higher in low income smokers [4, 5], which results in substantial health inequalities between higher- and lower- income groups [6].

Monitoring of national patterns of smoking and quitting behaviour is needed to inform policies and develop successful campaigns aimed at reducing tobacco-related harm. Relevant parameters to monitor include: smoking prevalence; rates, duration, trigger and success of quit attempts; exposure to health professionals’ advice on quitting; the use of harm reduction strategies such as “cutting down”; and the use of cessation aids [7]. Demographic information such as gender or socioeconomic status (SES) and the influence of potential confounders on the smoking cessation process, e.g., the motivation to stop or the degree of nicotine dependence, also have to be considered [7].

Several national large-scale surveys (ongoing or already completed) [4, 8–10] have been providing useful data on tobacco consumption and smoking behaviour in Germany, but they all have limitations. First, these surveys cover a wide range of addictions and other health topics in general which means that they do not allow for in-depth analysis of data on smoking behaviour. Second, these surveys collect data only on an annual or even less frequent basis. Data are therefore often out of date, and there is a lack of flexibility to adjust survey items to new, specific trends in smoking behaviour (e.g., the introduction of electronic (e-)cigarettes). This limits their topical relevance for tobacco control policies and research. Third, the sample sizes of some of these surveys are not sufficiently large to allow statistically reliable analyses.

Collection of observational “real-world” data on the effectiveness of smoking cessation methods is important to supplement randomised controlled trials (RCTs), whose generalisability is somewhat limited because of the participant selection criteria. There is good evidence from RCTs that behavioural support and pharmacological treatment, such as nicotine replacement therapy (NRT), bupropion, or varenicline, improve the success of quit attempts [11–15]. However, analyses on the real-world effectiveness of smoking cessation methods from the observational Smoking Toolkit Study (STS) from England [16] showed, for example, that smokers who buy NRT over the counter to aid their quit attempt have similar odds of success as those who tried to stop unaided [17], indicating the importance of providing cessation medication together with behavioural support. The STS is a large ongoing cross-sectional household survey which provides monthly data as well as six-month follow-up data on smoking and cessation behaviour in the English general population aged 16 years and older [16]. Findings from the STS cannot be directly transferred to the German health policy context. Whereas behavioural support and pharmacological smoking cessation treatment, for instance, is offered at no or minimal cost in England, such services do not exist in Germany, and evidence-based treatments are not or only partly reimbursed.

Moreover, little is known about the real-world effectiveness of e-cigarettes for smoking cessation in the German population. Its popularity and usage has rapidly increased in recent years. In England, for example, the prevalence of “ever-users” (people who used an e-cigarette at least once) in smokers and recent ex-smokers has increased from approximately 2% in 2011 to approximately 25% in 2016 [2]. Data from Germany show a comparable trend [3, 18]. Due to design and variety of flavours, e-cigarettes appear popular among adolescents. Authors have debated whether or not e-cigarettes may facilitate nicotine addiction in this age cohort [19, 20]. Therefore, tracking e-cigarette usage behaviour in adolescents is of particular importance.

### Aims

The DEBRA study will fill a crucial gap in the literature by collecting up-to-date and in-depth data on key indicators regarding the patterns and trends of smoking and quitting in the German population, helping to guide policy and clinical practice. The collection of baseline and follow-up data provides the possibility for longitudinal analyses on within-individual trends in relevant parameters. These data can contribute to a better understanding of smoking cessation processes in the general population and the role played by triggers such as motivation or physician advice, sociodemographic factors, and aids to cessation such as NRT, behavioural support, or even non-evidence-based methods, including e-cigarettes. The methodology of DEBRA is closely aligned to the STS, allowing comparisons with relevant data from the English population. The study design allows a flexible adjustment of the scope of the survey to address emerging questions relevant to policy changes, practice or research.

## Methods

### Design and sample

The DEBRA study started in June 2016 to collect data from computer-assisted face-to-face household interviews of people aged 14+. Over a period of 3 years, every two months, a new sample of approximately 2000 respondents will complete the survey (= 18 waves, approximately 36,000 respondents in total). Per wave, about 500–600 people are expected to smoke tobacco or to be recent ex-smokers (i.e., have smoked in the past 12 months). It is anticipated that data on approximately 10,000 smokers and recent ex-smokers will be collected during the 3-year period. All smokers and recent ex-smokers who are willing to be re-contacted will be followed-up at 6 months following baseline by telephone to complete a short survey. It is expected that about 120–150 (25%) from each baseline wave will agree to be followed-up, resulting in a total sample size of approximately 2500 respondents with baseline and follow-up data.

The fieldwork is conducted by the market research institute Kantar Health Munich, Germany. The investigation area is the Federal Republic of Germany. The total population of the study covers all German speaking persons aged 14 years or over who live in private households during the survey period. To keep the costs manageable, the baseline survey will use a multi-topic omnibus survey, a regular series of surveys from Kantar Health to which questions can be added. Per wave up to 280 trained interviewers will conduct on average 7 interviews each. Questions of the DEBRA study are placed in the middle of the 1 h multi-topic omnibus survey.

Baseline data collection will be facilitated through multi-stage, multi-stratified random probability sampling. Firstly, all communities within Germany will be stratified by the following regional attributes: federal state (*n* = 16), administrative districts (a maximum of *n* = 41 per federal state), and type of community (a maximum of *n* = 10 per district). For stratification purposes, these attributes will be used in combination. Hence, each type of community in every administrative district builds a different stratum, which theoretically leads to a total of 410, in practice fewer, strata.

To determine the primary sampling units for the study, the geographical area of Germany was divided into 53,000 small areas of approximately equal size, based on communities, and using geographic street mapping system software. For each small area, the sample size was determined in proportion to the total number of households in the area, relative to the total number of households in the whole population. This is called an area sample or sample point. In the current study the number of sample points varies between 360 and 410. All small areas were also allocated to their superordinate geographical units, such as rural and urban districts and municipalities, to allow further stratification of the sample. Sampling units of the second stage will be represented through private households and selected by a random walk procedure. Sampling units of the third stage will be the target persons themselves. These will be selected using a random process (so called “Schwedenschlüssel”), which gives an equal chance of selection to every eligible person within a household [21].

### Weighting techniques

Data analyses will take into account weighting adjustments for personal and household characteristics. According to the multi-stage sampling procedure of baseline data the weighting has to be done in separate stages, too. One has to differentiate between the so-called design weighting, which corrects unequal selection probabilities due to the sample design, and the outcome weighting, which corrects disproportions based on the structure of target persons who did or did not participate in the survey. Step 1 and step 3 (see further on) are calculated as rim-weighting within an iterative process. Step 2 is calculated by an analytical approach.

#### First step (outcome weighting)

The sampling procedure gives every household the same chance to be selected which means that the realised sample should be distributed proportionally to the distribution of private households. Within this first step, the structure of the realised sample is compared to the proportions of the original stratification of the first sampling stage. The weighting factors correct differences between the original and the realised household characteristics.

#### Second step (design weighting)

In sampling stage 3 (see below) every eligible person within a household has the same chance of selection. Hence, the selection probabilities of the target persons differ across the whole sample according to the size of the household they live in. This unequal selection probability is corrected by an analytical mathematical procedure using the size of the households. The household sample is changed into a sample of persons where every respondent has equal selection probability.

#### Third step (outcome weighting)

The last weighting step corrects for differences between the demographic structures of the respondents and the structures of the total population using the following structures: Federal states combined with sex and generally seven age classes.

### Baseline measures

The main baseline questionnaire for the DEBRA can be found in Additional file 1. To ensure comparability of data, the majority of questions has been adopted from the STS survey and labelled appropriately in the questionnaire [16]. STS questions were developed by an expert panel and policy makers [16]. Existing questions from the STS were translated into German and new questions were developed by a bilingual team of researchers (see authors’ contributions) with experience in using the STS data. Some of the validated scales from the STS were translated and culturally adapted according to international guidelines [22].

Baseline DEBRA questions for each respondent cover smoking status and ever-use of e-cigarettes. Depending on the response behaviour, current tobacco smokers (cigarettes or other tobacco products), recent ex-smokers (<12 months since quitting tobacco) and ever-users of e-cigarettes or a similar product (e.g., e-hookah, e-cigar, or e-pipe) will answer on further detailed questions about smoking behavior, quit attempts, exposure to health professionals’ advice on quitting, and use of cessation aids (Table 1). In addition, socioeconomic data (including age, sex, housing situation, residential area, marital status, education, respondent and household income, and employment status) will be regularly collected in all respondents as part of the omnibus survey.

**Table 1:** Content of the baseline questionnaire for current smokers, recent ex-smokers, and e-cigarette users

Current smokers and recent ex-smokers	1. Current smoking behaviour (or past smoking behaviour in those that have recently stopped)
	2. Current motivation to quit smoking using the Motivation to Stop Scale [27]
	3. Use of nicotine replacement therapy while smoking
	4. Current (or past) nicotine dependence using the Fagerström Test for Cigarette Dependence [29]
	5. Exposure to health professionals’ advice on quitting
	6. Current (or past) strength of urges to smoke [30]
	7. The number of serious quit attempts recalled as having been made within the past 12 months
	8. For the most recent quit attempt within the past 12 months: a. how long-ago the quit attempt started b. how long it lasted c. any aids used (e.g., nicotine replacement therapy over-the-counter, telephone helpline) d. what triggered it (external and internal triggers) e. whether it involved cutting down gradually f. and whether it was planned in advance [7]
E-cigarette users	1. Usage behaviour and its development
	2. Starting age and reasons for e-cigarette use
	3. Type, place of purchase and average consumption of liquid and nicotine in e-cigarettes

The study design is flexible. Depending on trends in population usage of tobacco or nicotine products, in tobacco research, or tobacco control policies, it is intended that new questions can be added from wave to wave while others will be removed when adequate statistical power is reached to address a specific scientific issue.

### Six-month follow-up

Smokers and recent ex-smokers who agree to be followed-up will be re-contacted by telephone six months following baseline. The computer assisted telephone interviews will be performed by interviewers from Kantar Healths’ call centre using a Sample Management System (SMS). The SMS manages and controls all telephone contacts (e.g., telephone numbers that cannot be contacted are set deferred, and presented again after a long interval at different times of a day). Interviewers will try to reach the respondents up to ten times at various times and on different days of the week.

Additional file 2 shows the main follow-up questionnaire for the DEBRA study. Follow-up questions focus on current smoking status and on detailed exploration of any quit attempts that have been made during the past six months. Methods used for a quit attempt (if so) will be asked in concordance to the baseline survey. This prospective element enables us to further analyse the real-world effectiveness of smoking cessation methods using longitudinal data [23] and providing data on within-individual trends. The short time frame between baseline and follow-up takes into consideration the fact that many smokers make multiple quit attempts within a short space of time and often rapidly forget unsuccessful ones [24]. Smokers who try to stop unaided, for example, seem to forget failed quit attempts more quickly than those who use treatment, what might lead to an underestimation of the effectiveness of stop-smoking medications [25].

### Main research topics addressed by the DEBRA study

The following research topics will be addressed from the beginning of data collection in wave 1 (June 2016). It is very likely that more topics will be added over time.

#### Prevalence rates and monitoring of trends

DEBRA will provide prevalence rates and proportions of key parameters on smoking status, smoking behaviour, quit attempts and their triggers, exposure to health professionals’ advice on quitting, and use of evidence-based as well as non-evidence-based cessation aids, including e-cigarettes, stratified by socioeconomic factors, nicotine dependence or motivation to quit. Longitudinal analyses from baseline to follow-up will provide insight into within-individual trends in each of these parameters. Time series analyses will assess changes including seasonal trends, linear trends, and as a function of policy changes or media campaigns.

#### Social gradient in quit attempts and successful abstinence

It is important to determine causes of the higher smoking prevalence rates in disadvantaged groups. Data from the STS showed that smokers in more deprived socioeconomic groups in England are just as likely as those in higher groups to try to stop and use aids to cessation [26]. However, there is a strong gradient across socioeconomic groups in success, with significantly lower success rates in disadvantaged groups being half as likely to succeed compared with the highest [26]. DEBRA data will examine to what extent these findings also apply to different socioeconomic groups in Germany.

#### External validation of the Motivation to Stop Scale (MTSS)

Smoker’s motivation to stop smoking is an important source of information, as it predicts the incidence of future quit attempts [27, 28]. Moreover, measuring changes in motivation to stop on a population level is useful to assess and to track the impact of tobacco control policies interventions over time. For this purpose, the single-item MTSS has been developed as a valid and cost-effective tool [27]. The MTSS combines key motivational constructs and has been shown to be a strong and accurate predictor of future quit attempts. With the DEBRA study, we aim to determine the external validity of the translated and culturally adapted MTSS (MRS: “Motivation zum Rauchstopp Skala”) among German smokers in predicting future quit attempts.

#### External validation of the Strength of Urges to Smoke Scale (SUTS)

An important criterion for a measure of nicotine dependence is how well it predicts relapse following smoking cessation. A commonly used scale to assess dependence and predict relapse is the Fagerström Test for Cigarette Dependence (FTCD) [29]. However, no representative data have been published on the predictive validity of the FTCD among German smokers. Furthermore, in recent ex-smokers, dependence cannot be measured with the FTCD, as one of its items includes the number of cigarettes smoked per day. By contrast, the two-item SUTS has been found to be a strong indicator of the severity of nicotine dependence and to be a better predictor of relapse than the FTCD [30] in the English population. Its use also allows analyses on the effectiveness of cessation methods (used during the last quit attempt) in recent ex-smokers through adjusting for urges to smoke as a confounder [17]. We aim to determine the external validity of the translated and culturally adapted SUTS (VRS: “Verlangen zu Rauchen Skala”) among German smokers and recent ex-smokers in measuring nicotine dependence and predicting relapse.

#### Real-world effectiveness of evidence-based and non-evidence-based smoking cessation methods, including e-cigarettes

Numerous analyses on the real-world effectiveness of smoking cessation aids have been conducted with data from the STS in England, for example, to supplement RCT data and to provide estimates for the effectiveness in the general population [17]. However, transferability of STS results to the German health policy context is difficult because evidence-based cessation aids are not or only partly reimbursed in Germany. The DEBRA study will provide such data for Germany. Moreover, hardly any national data is available on usage and real-world effectiveness of non-evidence-based methods, including e-cigarettes. In England, for instance, e-cigarettes have already become the most frequently chosen method of quitting tobacco, possibly contributing to the country’s decline in tobacco smoking [31]. The DEBRA study aims at assessing the emerging role of the e-cigarette as an aid in smoking cessation in Germany and evaluating its real-world effectiveness to complement sparse data coming from one existing RCT which found a clinically relevant difference in continuous 6-month abstinence between active vs. placebo e-cigarette users trying to quit [32].

#### Factors associated with the use of aids to cessation

Recent data from Germany suggest that only a small proportion of smokers use evidence-based aids while trying to quit [33]. Most smokers still try to quit unaided, which is associated with high relapse rates, particularly within the first weeks of abstinence where the strongest withdrawal symptoms occur [34]. The assessment of differences in sociodemographic or smoking characteristics might support health strategies to address specific groups of smokers who are underutilising evidence-based cessation aids in health strategies to a greater extent [35]. Therefore, the DEBRA study aims to analyse variations in use of smoking cessation aids among German smokers trying to quit.

#### Exposure to health professionals’ advice on quitting

Brief advice on smoking cessation delivered by a physician has been found to be effective and affordable [36, 37]. Hence, clinical practice guidelines recommend implementing brief advice on smoking cessation into routine primary care to assist a large number of smokers. Nevertheless, these recommendations seem to be only rarely implemented into practice in Germany [38] and hardly any representative and up-to-date data is available on frequency of delivery and approaches used to deliver such advice. The DEBRA study aims to provide such nationally representative data to inform policy health strategies aimed at implementing the clinical guidelines.

### Data analysis and sample size calculation

Statistical data analyses depend on scientific issues that will be addressed by the DEBRA study. Detailed analyses will therefore be reported in the publication of each future study, respectively.

The methodology of DEBRA is closely aligned to the English STS. Our assumptions about expected means and variances for the primary analyses are therefore made on the basis of these STS analyses [17, 23, 39, 40]. Data indicate that, depending on the specific issue, a sample between 1500 and 7000 interviewed smokers and recent ex-smokers are needed to yield adequate statistical power of >80%. The total required sample size was determined considering current prevalence rates of smokers in Germany [4], available data on rates of cessation attempts within the past 12 months [33], and physician visits within the past 12 months [41], whereas average response rates at follow-up were estimated by Kantar Health on the basis of previous surveys. During the 18 waves of the DEBRA study with approximately 2000 interviews per wave, about 500–600 people are expected to smoke tobacco or to be recent ex-smokers (in total = ~10,000). Based on these assumptions the required baseline sample sizes from a total of 1500 to 7000 smokers and recent ex-smokers can be achieved within 3 years. A response rate of 20–25% is expected at follow-up, resulting in a sample size of approximately 2500 after 3 years.

### Dissemination of findings

Findings from this survey will be reported in national and international peer-reviewed scientific journals, and at national and international conferences. Up-to-date statistics will be also published on the study website: www.debra-study.info.

## Discussion

Reducing the prevalence of tobacco smoking is one of the most important strategies for reducing the burden of morbidity, premature death, and health inequalities between higher and lower income groups within the next decades [42]. Continuous monitoring of national patterns of smoking and quitting behaviour as well as real-world data on the effectiveness of smoking cessation methods are needed to guide tobacco control policies and clinical practice.

The DEBRA study aims to provide such representative data for Germany. Its methodology is sufficiently flexible to allow adaptions of its research scope and tracking data on every two months permits a much more sensitive test of the possible effects of interventions than can be achieved by annual national surveys. Hence, DEBRA data will provide quick and direct estimates of policy or health strategy impacts aimed at reducing tobacco-related harm as well as of its cost-effectiveness.

The results of the DEBRA study can also be relevant to improve clinical care for smokers. Real-world data on the effectiveness of smoking cessation aids might complement the current state of knowledge on evidence-based and non-evidence-based smoking cessation processes which is usually obtained from RCTs. Data on prevalence of the use of such methods and on exposure to health professionals’ advice on quitting might help to analyse the as-is state of usage and care. Future interventional studies might then address discrepancies between the as-is (e.g., under-usage of evidence-based treatments to aid cessation) and the target state of use (e.g., use of evidence-based treatments). Such interventions could address both, the physicians and the patients themselves, to increase rates of recommendations as well as the use of evidence-based cessation aids.

We expect the DEBRA methodology to be robust and reliable. However, data from the first waves will have to be analysed to compare the representativeness of the DEBRA data to other ongoing national large scale surveys [4, 8–10]. Slight deviations can certainly occur due to different modes of data collection (face-to-face interview, telephone, or online), seasonal fluctuations, differences in weighting procedures, as well as differences due to the inclusion of diverse age groups of participants.

There are certain general limitations that apply to large national surveys such as the DEBRA study, including those associated with self-report which may be biased or inaccurate. Furthermore, drop-out rates from baseline to six month follow-up are expected to be substantial and possibly differential. However, representativeness of the follow-up samples will be examined in detail by comparisons with baseline data on key variables from those not followed up and results from such analyses will be interpreted accordingly. Finally, even if the DEBRA study aims to collect a sample of smokers large enough to yield adequate statistical power for several subgroup analyses, certain analyses are likely to be underpowered due to small sample sizes, e.g. in relation to ethnicity, specific professions, or usage of specific e-inhalation products (e.g., e-hookah, e-cigar, or e-pipe).

## Conclusions

The DEBRA study will enhance understanding of factors influencing smoking and cessation behaviour on a population level and be an important resource of scientific data for the development of tobacco control policies, health strategies, and future studies in Germany. The study design allows a flexible adjustment of the scope of the survey to specific trends in smoking behaviour, topical questions in tobacco research or policies. The methodology of the study is closely aligned to the STS, allowing international comparisons of data.

## Additional files

Additional file 1: DEBRA - Baseline survey. (DOCX 66 kb)

Additional file 2: DEBRA - Follow-up survey. (DOCX 38 kb)

## Abbreviations

DEBRA: German Study on Tobacco Use (“Deutsche Befragung zum Rauchverhalten”)
DRKS: German Clinical Trials Register (“Deutsches Register Klinischer Studien”)
E-cigarettes: Electronic cigarettes
FTCD: Fagerströmtest Test for Cigarette Dependence
MTSS: Motivation to Stop Scale
NRT: Nicotine replacement therapy
RCT: Randomised controlled trial
SES: Socioeconomic status
SMS: Sample Management System
STS: Smoking Toolkit Study
SUTS: Strength of Urges to Smoke Scale

## References

[1] Mons U. Tobacco-attributable mortality in Germany and in the German Federal States – calculations with data from a microcensus and mortality statistics.[Article in German] Gesundheitswesen. 2011;73:238-246. 10.1055/s-0030-1252039 20549599

[2] West R, Brown J. Latest trends on smoking in England from the Smoking Toolkit Study. http://www.smokinginengland.info/sts-documents/. Accessed 16 Mar 2017.

[3] European Commission (2015). Special Eurobarometer 429: Attitudes of Europeans towards tobacco and electronic cigarettes.

[4] Lampert T, von der Lippe E, Muters S. Prevalence of smoking in the adult population of Germany: results of the German Health Interview and Examination Survey for Adults (DEGS1). [Article in German] Bundesgesundhbl Gesundheitsforsch Gesundheitsschutz. 2013;56:802-808. 10.1007/s00103-013-1698-1 23703501

[5] Lampert T. Social determinants of tobacco consumption among adults in Germany. [Article in German].Bundesgesundhbl Gesundheitsforsch Gesundheitsschutz. 2010;53:108-116. 10.1007/s00103-009-1014-2 20076933

[6] Mackenbach JP, Stirbu I, Roskam A-JR, Schaap MM, Menvielle G, Leinsalu M (2008). Socioeconomic Inequalities in Health in 22 European Countries. New Engl J Med..

[7] West R. Smoking toolkit study: protocol and methods. http://www.smokinginengland.info/sts-documents/. Accessed 26 Jan 2017.

[8] Lampert T, Kuntz B. Tobacco and alcohol consumption among 11- to 17-year-old adolescents: results of the KiGGS study: first follow-up (KiGGS Wave 1) [Article in German]. Bundesgesundhbl Gesundheitsforsch Gesundheitsschutz. 2014;57:830-839. 10.1007/s00103-014-1982-8 24950832

[9] Robert Koch-Insitut. Daten und Fakten: Ergebnisse der Studie »Gesundheit in Deutschland aktuell 2012«. Beiträge zur Gesundheitsberichterstattung des Bundes. [Article in German]. 2014. http://www.rki.de/DE/Content/Gesundheitsmonitoring/Gesundheitsberichterstattung/GBEDownloadsB/GEDA12.html. Accessed 06 Mar 2017.

[10] Piontek D, Kraus L,Gomes de Matos E, Atzendorf J. Der Epidemiologische Suchtsurvey 2015: Studiendesign und Methodik [Article in German]. Sucht. 2016;62:259–269.

[11] Lancaster T, Stead LF. Individual behavioural counselling for smoking cessation. Cochrane Database Syst Rev. 2005;18(2):CD001292. 10.1002/14651858.CD001292.pub2 15846616

[12] Stead LF, Buitrago D, Preciado N, Sanchez G, Hartmann-Boyce J, Lancaster T. Physician advice for smoking cessation. Cochrane Database Syst Rev. 2013;31(5):CD000165. 10.1002/14651858.CD000165.pub4 PMC7064045 23728631

[13] Stead LF, Lancaster T. Group behaviour therapy programmes for smoking cessation. Cochrane Database Syst Rev. 2005;18(2):CD001007. 10.1002/14651858.CD001007.pub2 15846610

[14] Cahill K, Lindson-Hawley N, Thomas KH, Fanshawe TR, Lancaster T. Nicotine receptor partial agonists for smoking cessation. Cochrane Database Syst Rev. 2012;18(4):CD006103. 10.1002/14651858.CD006103.pub7 PMC6464943 27158893

[15] Stead LF, Koilpillai P, Fanshawe TR, Lancaster T (2016). Combined pharmacotherapy and behavioural interventions for smoking cessation. Cochrane Database Syst Rev..

[16] Fidler JA, Shahab L, West O, Jarvis MJ, McEwen A, Stapleton JA (2011). 'The smoking toolkit study': a national study of smoking and smoking cessation in England. BMC Public Health..

[17] Kotz D, Brown J, West R (2014). 'Real-world' effectiveness of smoking cessation treatments: a population study. Addiction..

[18] Eichler M, Blettner M, Singer S (2016). The Use of E-Cigarettes. Dtsch Arztebl Int..

[19] Farsalinos KE, Romagna G, Tsiapras D, Kyrzopoulos S, Spyrou A, Vouthis V (2013). Impact of flavour variability on electronic cigarette use experience: an internet survey. Int J Env Res Pub He..

[20] Brown J (2017). A gateway to more productive research on e-cigarettes? Commentary on a comprehensive framework for evaluating public health impact. Addiction..

[21] Kish L (1949). A Procedure for Objective Respondent Selection within the Household. JASA..

[22] Wild D, Grove A, Martin M, Eremenco S, McElroy S, Verjee-Lorenz A (2005). Principles of good practice for the translation and cultural adaptation process for Patient-Reported Outcomes (PRO) measures: report of the ISPOR Task Force for Translation and Cultural Adaptation. Value Health..

[23] Kotz D, Brown J, West R (2014). Prospective cohort study of the effectiveness of smoking cessation treatments used in the “real world”. Mayo Clin Proc..

[24] West R. Feasibility of a national longitudinal study (‘The Smoking Toolkit Study’) to monitor smoking cessation and attempts at harm reduction in the UK. http://www.smokinginengland.info/sts-documents/ (Ref: STP001). Accessed 06 Feb 2017.

[25] Borland R, Partos TR, Cummings KM (2012). Systematic biases in cross-sectional community studies may underestimate the effectiveness of stop-smoking medications. Nicotine Tob Res..

[26] Kotz D, West R (2009). Explaining the social gradient in smoking cessation: it's not in the trying, but in the succeeding. Tob Control..

[27] Kotz D, Brown J, West R (2013). Predictive validity of the Motivation To Stop Scale (MTSS): a single-item measure of motivation to stop smoking. Drug Alcohol Depend..

[28] Ussher M, Kakar G, Hajek P, West R (2016). Dependence and motivation to stop smoking as predictors of success of a quit attempt among smokers seeking help to quit. Addictive Behaviors..

[29] Fagerström K (2012). Determinants of tobacco use and renaming the FTND to the Fagerstrom Test for Cigarette Dependence. Nicotine Tob Res..

[30] Fidler JA, Shahab L, West R (2011). Strength of urges to smoke as a measure of severity of cigarette dependence: comparison with the Fagerstrom Test for Nicotine Dependence and its components. Addiction..

[31] Public Health England. E-cigarettes: an evidence update. A report commissioned by Public Health England 2015. https://www.gov.uk/government/uploads/system/uploads/attachment_data/file/457102/Ecigarettes_an_evidence_update_A_report_commissioned_by_Public_Health_England_FINAL.pdf. Accessed 27 Mar 2017.

[32] Bullen C, Howe C, Laugesen M, McRobbie H, Parag V, Williman J (2013). Electronic cigarettes for smoking cessation: a randomised controlled trial. Lancet..

[33] Kröger CB,Gomes de Matos E, Piontek D, Wenig JR Quitting attempts and utilisation of smoking cessation aids among smokers in Germany: results from the 2012 Epidemiological Survey of Substance Abuse.[Article in German] Gesundheitswesen. 2016;78:752-758. 10.1055/s-0035-1548913 26158343

[34] Hughes JR, Keely J, Naud S (2004). Shape of the relapse curve and long-term abstinence among untreated smokers. Addiction..

[35] Kotz D, Fidler J, West R (2009). Factors associated with the use of aids to cessation in English smokers. Addiction..

[36] Stead L, Bergson G, Lancaster T. Physician advice for smoking cessation. Cochrane Database Syst Rev. 2008;16(2):CD000165. 10.1002/14651858.CD000165.pub3 18425860

[37] West R, Raw M, McNeill A, Stead L, Aveyard P, Bitton J (2015). Health-care interventions to promote and assist tobacco cessation: a review of efficacy, effectiveness and affordability for use in national guideline development. Addiction..

[38] Twardella D, Brenner H (2005). Lack of training as a central barrier to the promotion of smoking cessation: a survey among general practitioners in Germany. Eur J Public Health..

[39] Kotz D, Brown J, West R (2014). Prospective cohort study of the effectiveness of varenicline versus nicotine replacement therapy for smoking cessation in the "real world". BMC Public Health..

[40] Brown J, Beard E, Kotz D, Michie S, West R (2014). Real-world effectiveness of e-cigarettes when used to aid smoking cessation: a cross-sectional population study. Addiction..

[41] Grobe TG, Steinmann S, Szecsenyi J. Barmer GEK's report. [Article in German]. https://www.barmer.de/blob/36738/41528a9e5704bb8d47e25e00707af4ba/data/pdf-arztreport-2016.pdf. Accessed 19 Mar 2017.

[42] U.S. Department of Health and Human Services. The health consequences of smoking - 50 years of progress: a report of the Surgeon General 2014. https://www.surgeongeneral.gov/library/reports/50-years-of-progress/full-report.pdf. Accessed 04 Feb 2017.

